# Treatment of Complex Wounds with NovoSorb^®^ Biodegradable Temporising Matrix (BTM)—A Retrospective Analysis of Clinical Outcomes

**DOI:** 10.3390/jpm12122002

**Published:** 2022-12-03

**Authors:** Frederik Schlottmann, Doha Obed, Alperen S. Bingöl, Vincent März, Peter M. Vogt, Nicco Krezdorn

**Affiliations:** Department of Plastic, Aesthetic, Hand and Reconstructive Surgery, Hannover Medical School, Carl-Neuberg-Strasse 1, 30625 Hannover, Germany

**Keywords:** wound therapy, dermal skin templates, skin reconstruction, complex wounds, *Pseudomonas aeruginosa*, NovoSorb^®^ Biodegradable Temporising Matrix, BTM, plastic-reconstructive surgery

## Abstract

Complex and chronic wounds represent a highly prevalent condition worldwide that requires a multimodal and interdisciplinary treatment approach to achieve good functional and aesthetic outcomes. Due to increasing costs of health care, an aging population and an increase in difficult-to-treat microbial colonization of wounds, complex wounds will become a substantial clinical, social and economic challenge in the upcoming years. In plastic reconstructive surgery, a variety of dermal skin substitutes have been established for clinical use. Since its approval as a dermal skin substitute in Germany, NovoSorb^®^ Biodegradable Temporising Matrix (BTM) has become a valuable therapeutic option for the treatment of full-thickness wound defects. The clinical data published to date are limited to case reports and small-scale case series with the main focus on single wounds. The aim of this single-center study was a retrospective analysis of our own patient collective that has received treatment with BTM for complex wounds. Overall, BTM showed to be a reliable and versatile reconstructive option, especially for patients with multiple co-morbidities and microbiologically colonized wounds. Although the preliminary findings have produced promising results, further investigation and research are warranted regarding long-term outcomes and additional clinical applications.

## 1. Introduction

The skin, as one of the largest organs of the human body, plays a crucial role in maintaining the organism’s function. It has a key role as a mechanical and chemical barrier to the environment and thereby prevents fluid and thermal loss [1,2]. Damage to the skin, especially in the context of complex wounds, still poses a major challenge to the plastic reconstructive surgeon [3]. It is necessary to distinguish between “simple” wounds, for example, surgical wounds or skin scratches, and “chronic” or “complex” wounds that do not heal primarily and require specialized treatment, mostly in the context of inpatient treatment [4]. The following proposed definition by Ferreira et al. [4] identifies four criteria, which have to be met, in order to label a wound as “complex”:

1. Extensive loss of the integument in acute or chronic wounds (chronic wounds are defined as non-spontaneously healing wounds within 3 months [5]);
2. Infection, as a complication in chronic wounds, can cause tissue loss (e.g., aggressive infections such as Fournier’s gangrene);
3. Necrosis and compromised viability of surrounding tissue or signs of circulation impairment;
4. Systematic pathologies that impair normal wound healing (e.g., diabetes, vasculitis or immune suppression).

Based on the criteria of Ferreira et al. the following wounds can be considered “complex” wounds: burns, full-thickness wounds in the lower extremity, diabetic ulcers, pressure ulcers, chronic venous ulcers, wounds following extensive necrotic processes caused by infections, such as Fournier’s gangrene, or necrotizing fasciitis [4]. These complex wounds have a significant impact on the patient’s personal life with a reduced quality of life due to chronic pain, reduced mobility, regular visits to the doctor for wound treatment or even inpatient treatment, often lasting for several weeks [3,6]. In addition to the personal impact, complex wounds represent a significant socioeconomic burden with increasing overall importance. In the United Kingdom (UK), studies have calculated the costs for the treatment of complex wounds to the National Health Service (NHS) to around £1 billion yearly [5]. For example, diabetic foot ulcerations in the UK resulted in 24,000 hospital admissions, costing the NHS around £17 million [7] whereas the management of these complex wounds was estimated to cost $150 million yearly in the United States (US) [8]. Apart from the socioeconomic burden, chronic wounds have a considerable impact on the patient’s quality of life leading to immobility, psychosocial stress and chronic pain [9]. Due to increasing costs of health care, an aging population and an increase in difficult-to-treat microbial colonization of wounds, complex wounds will become a substantial clinical, social and economic challenge in the upcoming years. It is estimated that the annual wound care product market will reach $15–22 billion by 2024 [10].

Concomitant pre-existing conditions and comorbidities often complicate the management of patients with complex wounds [11]. Taking into account systemic underlying diseases and comorbidities such as arterial hypertension, diabetes, immunological and rheumatological diseases, as well as peripheral arterial occlusive disease, the treatment of this patient group is very complex and requires an interdisciplinary approach [3]. Creating independent multidisciplinary wound centers, such as The Copenhagen Wound Healing Center (Copenhagen, Denmark) and the University Center of Wound Healing (Odense, Denmark), has resulted in improved rates of healing in patients with leg ulcers and reduced rates of major amputations [12]. The structure of specialized wound centers offers manifold opportunities for basic and clinical research and provides differentiated training for all types of healthcare professionals [12].

In the complex and multimodal approach to the treatment of complex wounds, a distinction must be made between conservative and surgical therapeutic procedures [13]. Reconstructive surgical options for covering complex skin soft tissue defects encompass the entire spectrum of plastic reconstructive techniques. Epidermal or partial dermal skin soft tissue defects can ideally be covered by autologous split-thickness skin grafting [14]. Autologous split-thickness skin grafting has proven to be a safe, low-risk, and well-established method of defect coverage when treating patients with complex wounds and multiple comorbidities [15,16]. Allogeneic skin grafts also have a place in the care of severely burned patients to a certain extent and are widely used for wound management in burn centers [17]. However, donor site morbidity of autologous skin grafts and graft rejection of allografts limit their clinical applications [18]. Especially in deep dermal complex wounds, skin grafting is often no longer sufficient to cover the defect, so dermal skin substitutes are used in clinical routine [19]. There is a plethora of different dermal skin substitutes commercially available on the market with the spectrum ranging from biological to completely synthetic skin substitutes [20]. Overall, dermal skin substitutes can be classified according to their composition, e.g., decellularized dermis derived from human or animal sources, artificially constructed scaffolds comprised of highly purified biomaterials or entirely synthetic polymers [18]. Table 1 lists examples of some of the dermal skin substitutes currently available. The list should only be seen as an excerpt of the diversity of available dermal skin substitutes and is based on an earlier publication by the authors [18].

Since its approval as a dermal skin substitute in Germany, BTM has become a valuable therapeutic option for the treatment of full-thickness wound defects [27]. According to the manufacturer’s specifications, BTM is a fully synthetic dermal skin matrix for the reconstruction of complex wounds that does not contain any biological molecules. The wound bed facing 2 mm thick NovoSorb^®^ biodegradable polyurethane cell foam is covered by a non-biodegradable sealing membrane [28]. The porous matrix allows cell migration and proliferation throughout the whole matrix and thereby neo-angiogenesis and vascularization [28]. At the same time, it functions as a scaffold for the emerging neo-dermis [28]. The first capillary refill could be observed as early as 2 weeks after defect coverage [29]. As the remodeling processes of the wound progress, hydrolytic degradation of the BTM matrix occurs [29]. The non-biodegradable sealing membrane provides a mechanical and physiological wound barrier and allows secretion drainage of the wound bed through the small fenestrations present [30]. BTM is used in a two-stage operative procedure. In the first stage, BTM is placed on a clean wound bed followed by a second operation with the removal of the sealing membrane and split-skin grafting on the neo-dermis [30]. This two-stage approach usually extends over a treatment period of up to 3 weeks [30]. Early in vivo studies have proven that BTM showed a reasonable biocompatibility that eliminates the possibility of interspecies immune rejection or disease transmission by being a fully synthetic material [26,29,31]. Rat and porcine models demonstrated the sufficient reconstruction of full-thickness skin defects with the absence of systemic toxic effects or wound contracture [29,31]. Furthermore, the use of BTM omits ethical and cultural obstacles associated with skin substitutes of human and animal origin [32]. The use of BTM in burns showed thought-provoking results as it was used to successfully treat large total body area burns with reliable and appealing aesthetic and functional outcomes [33,34]. In addition to its use in the treatment of burns, BTM also showed convincing functional and aesthetic results in the treatment of complex wounds after necrotizing fasciitis [35], for defect reconstruction after trauma [36] and for defect coverage of the donor site defects after free micro-vascular flaps [30,37].

The clinical data published to date are limited to case reports and small-scale case series and mainly focus on a single wound entity. Therefore, the aim of the present study was a retrospective analysis of the entire patient population at the Department of Plastic, Aesthetic, Hand and Reconstructive Surgery at Hannover Medical School (Hannover, Germany) that has received treatment with BTM for complex wounds, including patients with severe concomitant diseases and infected wound situations.

## 2. Materials and Methods

### 2.1. Patient Population

The study was designed as a single-center retrospective analysis of patients who underwent complex wound treatment with BTM. Other reconstructive surgeries and more complex surgical procedures were not initially considered because of pre-existing comorbidities. The study was conducted according to the guidelines of the Declaration of Helsinki and approved by the Ethics Committee of Hannover Medical School (protocol code: 9947_BO_K_2021 and date of approval: 11 August 2021). All patients gave informed consent for the analysis and processing of their treatment data in this study. Over the period from January 2020 to April 2021, 20 patients were treated with BTM at the Department of Plastic, Aesthetic, Hand and Reconstructive Surgery at Hannover Medical School. Twelve of them were male and eight were female. Patient demographics, indications for BTM, surgical details, co-morbidities as well as microbial data were included in the analysis. Patients with residual malignancy were excluded. The localization of the complex wound was recorded separately and divided into the following categories: lower extremity, upper extremity, trunk, head, neck and genital. Exemplary case reports of patients treated with BTM are presented in detail under Section 3.1, Section 3.2, Section 3.3, Section 3.4 and Section 3.5.

### 2.2. NovoSorb^®^ BTM Matrices and Operative Procedures

The majority of patients initially underwent radical surgical wound debridement, and the wound was initially temporarily dressed and conditioned with vacuum dressing with a continuous suction of 125 mmHg. A 3M™ V.A.C.^®^ system was used according to the manufacturer’s instructions (3M™ + KCI, St. Paul, MN, USA). Only the patient with neurofibromatosis and the patients with unstable scars and keloids primarily received BTM transplantation directly. Once the wounds were macroscopically clean and infection-free, BTM was applied. BTM was procured commercially (Polymedics, Denkendorf, Germany). All procedures were performed under general anesthesia. The BTM was initially fixed to the wound bed with staples or sutures. The edges of the wound were partially sutured down in some cases to improve dermal contact. According to the manufacturer’s instructions, the transplanted BTM was covered again by vacuum dressing over fetty gauze. Vacuum therapy was continued for 21 days, with a change of vacuum dressing every 7 to 10 days under general anesthesia. During the surgical procedures, vascularization and vascular sprouting of the BTM were assessed by macroscopic criteria and increasingly visible recapillarization. If vascularization of the BTM matrix was visible, delamination was performed according to the manufacturer’s instructions and definitive defect coverage was performed using autologous split-thickness skin grafting. According to our departmental standard, an overknot dressing was applied for 5 days. The time points of BTM application to final defect coverage by autologous split-thickness skin grafting were also determined. A take rate of the BTM of more than 90% was defined as healing. Smaller residual defects were subjected to secondary wound healing. Endpoints were defined as the integration of the BTM, loss of the BTM with necessary secondary complex reconstructive surgery and death during the course of treatment.

### 2.3. Microbial Analysis

Prior to surgical wound disinfection as part of the surgical procedure of the BTM transplantation, wound swabs were taken to assess the microbiological colonization of the wound. A total of 20 wound swabs from all 20 patients were included in the evaluation. A distinction was made between monobacterial colonization, mixed flora and sterile wound swabs. The pathogens identified were collected in a table and presented graphically using Microsoft Excel for Mac 2011 software (Version 14.6.9) (Microsoft Corporation, Redmond, WA, USA).

### 2.4. Clinical Outcome

Photographic documentation of the wounds was performed prior to discharge from inpatient hospital treatment. In the further course of treatment, further photo documentation took place when the patients were seen within our outpatient clinic. The follow-up as well as possible complications occurring during the course of treatment were determined.

## 3. Results

**Figure 1:**
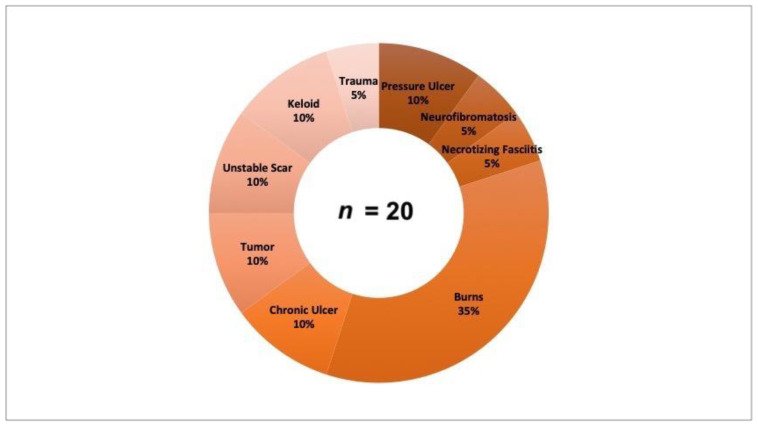
Entities of complex wounds and the relative proportions of each entity in the total population (*n* = 20).

All patients treated with BTM during the study period of 16 months, from January 2020 to April 2021, were included in the analysis. A total of 20 patients were included in this monocenter study. Of the included patients, 8 were female and 12 were male. The median age was 50.8 years and ranged from 15 to 82 years. The indication for the use of BTM took into account the existing concomitant diseases, some of which, due to their severity, represented a contraindication for a complex surgical procedure. The entities of the full-thickness skin defects of the complex wounds varied and are shown in Figure 1. In total, two pressure ulcers, one case of neurofibromatosis, one necrotizing fasciitis, seven burns, two chronic ulcers, two defects after tumor resection, two unstable scars, two full-thickness soft tissue defects after keloid resection and one full-thickness soft tissue defect after trauma were included.

The localization of the included complex wounds covered the entire body and included in total 27 complex wounds in 20 patients. The majority of the wounds (40.7%) were localized in the lower extremity. Table 2 shows in detail the localization of the 27 complex wounds.

The majority of patients initially underwent radical surgical wound debridement, and the wound was initially temporarily dressed and conditioned with vacuum dressing. Only the patient with neurofibromatosis and the patients with unstable scars and keloids primarily received BTM transplantation directly. After removal of the avital and infected tissue, vacuum conditioning of the complex wounds showed macroscopically clean wound conditions, so the indication for defect coverage using BTM was given. In the case of malignancies, oncological clearance was obtained histopathologically prior to BTM reconstruction.

The microbiological samples for the present study were collected during the operation in which the BTM was transplanted. All wounds were considered to be clinically infection-free when the BTM transplantation occurred. According to a standardized procedure, all wounds were swabbed intraoperatively prior to surgical disinfection. In the microbiological wound swabs, nine wounds showed an isolated pathogen, whereas nine wounds showed colonization with a mixed flora. Only two wound swabs were found to be sterile. A total of eight different pathogen strains were detected, with *Staphylococcus* ssp. and *Pseudomonas* ssp. being the most common. Table 3 shows the entire pathogen spectrum with the corresponding prevalence.

**Figure 2:**
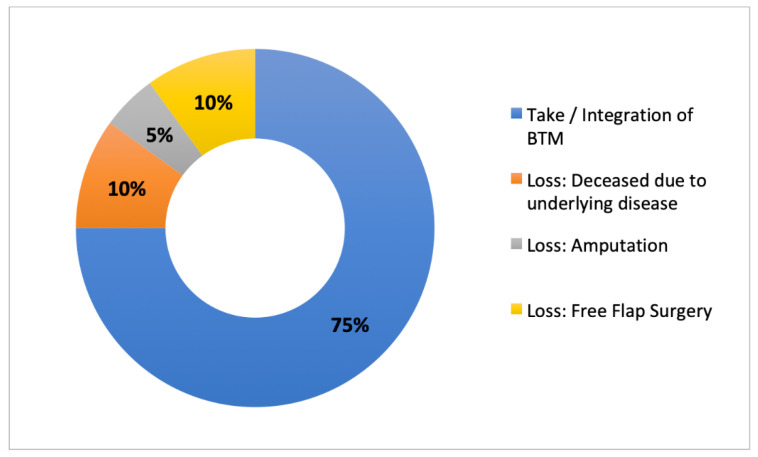
Overview of integration of NovoSorb^®^ Biodegradable Temporising Matrix (BTM) in the treatment of complex wounds (*n* = 20).

BTM was applied to the wounds in a multistage surgical procedure. After the initial application of BTM to the clean wound bed, BTM was fixed with staples or sutures. The average time from BTM transplantation to definitive defect coverage by autologous split-thickness skin grafting was 23.5 days, ranging from 13 to 49 days. During the entire treatment period, the wounds were conditioned using vacuum dressings. All patients remained hospitalized during the treatment period. Integration of BTM was assessed by macroscopically visible vascularization and recapillarization. Overall, BTM was integrated into 15 of 20 patients. Figure 2 shows the overall integration results of BTM in complex wounds.

Two patients died due to extensive burn injury and resulting multi-organ failure and septic shock before definitive defect coverage by autologous split-thickness skin grafting could be performed. One patient with a chronic ulcer lost the transplanted BTM due to a lack of integration. Because of the disastrous peripheral perfusion of the affected leg and the patient’s wish for a low-risk solution, a transtibial amputation with prosthetic treatment was performed in this case. Furthermore, a patient with a third-degree contact burn of the foot and co-existing diabetes mellitus also showed loss of BTM. In this case, defect coverage was performed using free lattissimus dorsi muscle flap plasty. In another case, a pressure ulcer covered with BTM also showed a lack of integration, so defect coverage by means of local transposon flap plasty was necessary. In the following, five clinical cases are presented as examples.

### 3.1. Case 1: Treatment of a Complex Wound after Necrotizing Fasciitis with BTM

**Figure 3:**
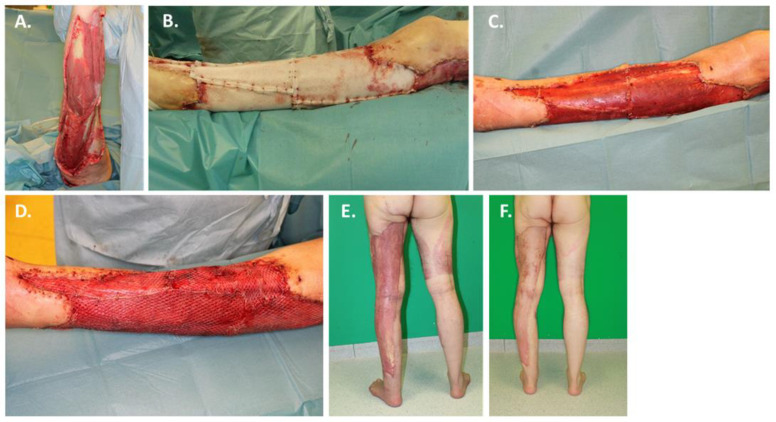
A case of necrotizing fasciitis treated with BTM. A 56-year-old male was admitted with necrotizing fasciitis affecting semicircular the left lower leg and the left thigh dorsal to gluteal: (**A**) Wound situation after multiple surgical debridement for local remediation; (**B**) At the time of implantation of BTM *Klebsiella pneumoniae* could be detected microbiologically; (**C**) Successful integration and delamination of BTM after 20 days; (**D**) Definitive defect coverage with autologous split-thickness skin grafting; (**E**) 3-month follow-up; (**F**) 6-month follow-up.

A 56-year-old male was referred to us from a peripheral hospital for further surgical therapy following necrotizing fasciitis. The left lower leg was affected semi-circularly and the left thigh dorsally to gluteally by the severe infection (Figure 3A). The patient had no known secondary diseases. Several surgical debridement as well as wound conditioning by means of vacuum dressing were performed under general anesthesia. When macroscopically clean wound conditions were achieved, the extensive skin soft tissue defects were covered with BTM (Figure 3B). Further wound conditioning was performed with vacuum dressings until visible vascularization and peripheral recapillarization of the BTM were observed. At the time of BTM transplantation, *Klebsiella pneumoniae* was detected in the wound swabs. The patient did not receive antimicrobial therapy at the time of BTM transplantation. After 20 days, definitive defect coverage with autologous split-thickness skin grafting with harvesting from the thigh of the contralateral side could be performed (Figure 3C,D). The BTM integrated completely. In a follow-up after 3 (Figure 3E) and 6 months (Figure 3F), the patient was completely mobile again and showed a satisfactory scar pattern. The patient was able to resume competitive sports.

### 3.2. Case 2: Treatment of a Sacral Pressure Ulcer with BTM

**Figure 4:**
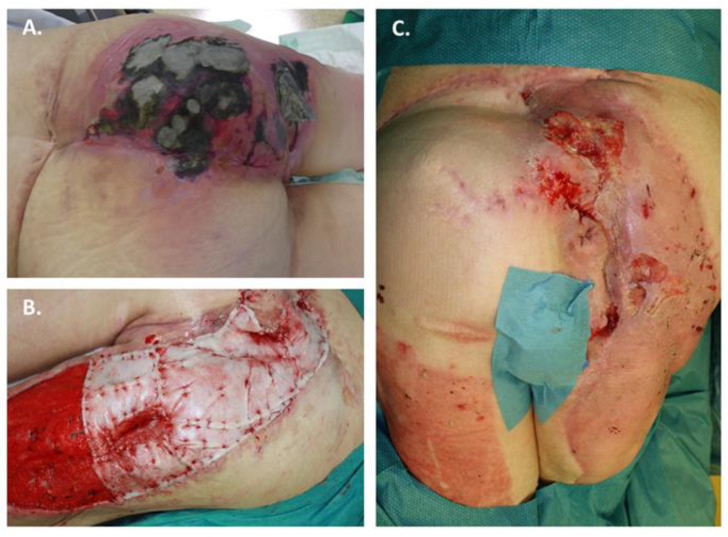
A case of pressure ulcer treated with BTM. A 47-year-old female was admitted with an extensive skin soft tissue defect in the gluteal and sacral region due to a pressure ulcer: (**A**) Wound situation on admission; (**B**) At the time of BTM implantation *Pseudomonas aeruginosa*, *Enterococcus faecalis* and *Staphylococcus aureus* were detected microbiologically; (**C**) Wound situation after removal of the overknot dressing with a loss of the split-skin and BTM on an area of 10%.

A 47-year-old female was presented to us with an extensive skin soft tissue defect in the gluteal and sacral region due to a pressure ulcer that had been present for several months (Figure 4A). The skin soft tissue defect affected mainly the right buttock half and the right dorsal thigh. The cause of the ulcer was spina bifida paraplegia with a sensitivity caudal to the lumbar region and resulting immobility. In her medical history, multiple local flap plasties and autologous split skin transplantations had already been performed to cover the defect, but all procedures failed. The secondary diagnosis was arterial hypertension. Multiple surgical procedures were performed using radical surgical debridement and wound conditioning using vacuum dressings. Histopathological evidence of osteomyelitis of the partially ablated os sacrum was also found. Due to the exhausted local defect coverage possibilities, the indication for BTM was given. At the time of BTM transplantation, microbiological evidence of *Pseudomonas aeruginosa*, *Enterococcus faecalis* and *Staphylococcus aureus* was found. The patient did not receive antimicrobial therapy at the time of BTM transplantation. The wound covered with BTM was sealed with vacuum dressings until definitive defect coverage could be achieved after 22 days using autologous split-thickness skin grafting (Figure 4B). After the removal of the overknot dressings, the split-skin grafts were 90% healed. In the further course of treatment, a wound healing disorder with loss of the BTM and the autologous split skin was observed in the sacral region, so the indication for BTM transplantation was given again (Figure 4C). After 13 days, however, the BTM did not adhere to the wound bed, so it was removed, and the wounds were subjected to secondary wound healing. Under conservative measures, the residual defects were healed, and the patient was discharged for further outpatient treatment. In a follow-up after 6 months, despite all positioning measures and aids, a new sacral decubitus ulcer measuring approximately 4 × 5 cm was found. The wounds were again subjected to conservative wound treatment.

### 3.3. Case 3: Treatment of an Unstable Scar after Early Childhood Burn with BTM

**Figure 5:**
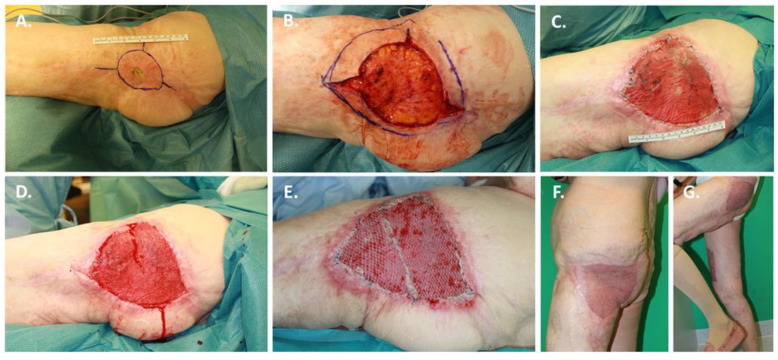
A case of an unstable scar after early childhood burn treated with BTM. A 79-year-old female was presented to us with an early childhood scald on the left buttock and thigh, which she had suffered at the age of 3 years: (**A**) Wound situation on admittance; (**B**) Wound situation for implantation of BTM; (**C**) Vascularized BTM after 21 days; (**D**) BTM after delamination; (**E**) Definitive defect coverage with autologous split-thickness skin grafting; (**F**,**G**) 3-month follow-up with appealing aesthetic and functional results.

A 79-year-old female was presented to us with an early childhood scald on the left buttock and thigh, which she had suffered at the age of 3 years. There had been no surgical therapy for the wounds after the trauma. Hypertrophic scars had formed in this area, which had been excised at another hospital. In the course of time, recurrent wound healing disorders had occurred, so that a partial resection of the complex wounds and a defect closure by means of advancement flap plasty had been performed on an unstable scar area. Since then, recurrent wound healing disorders occurred, which persisted under conservative therapy measures. The clinical examination revealed an unstable, but partially non-irritant scar plate in the area of the entire left lateral thigh with extensions into the gluteal region on the left side. Centrally over the trochanter region, a 3 × 1 cm wound healing defect with a fibrin-covered wound bed was visible (Figure 5A). There was a clear calcification in the distal wound area, which was also pressure-dolent. Proximal to the already described wound healing disorder a new porus was visible, but there was no evidence of fulminant wound infection. Secondary diagnoses were atrial fibrillation with anticoagulant therapy with apixaban and frequency control with beta-blockers.

Due to the exhausted local defect coverage possibilities, the indication for BTM was given (Figure 5B). At the time of BTM transplantation, microbiological evidence of *Pseudomonas aeruginosa* was found. The patient did not receive antimicrobial therapy at the time of BTM transplantation. The wound covered with BTM (Figure 5C) was sealed with vacuum dressings until definitive defect coverage could be achieved after 21 days using autologous split-thickness skin grafting (Figure 5D,E). After the removal of the overknot dressings, the split-skin grafts were 100% healed (Figure 5F). In a follow-up after 3 months, the patient was completely mobile again and showed a satisfactory scar pattern and showed self-sufficiency in domesticity (Figure 5G).

### 3.4. Case 4: Treatment of Superinfected and Non-Healing Wounds after Burn with BTM

**Figure 6:**
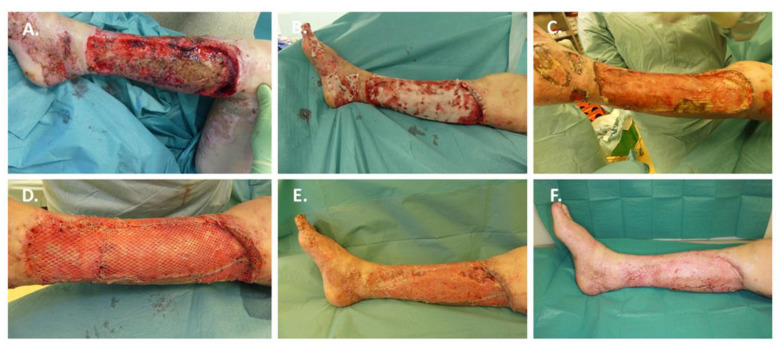
A case of superinfected and non-healing wounds after burn treated with BTM. A 69-year-old female was admitted primarily for the care of extensive lower extremity burn injuries bilaterally. A total of 15% of the total body surface area was burned 2a-3°. (**A**) Wound situation with recurrent wound infections with multi-resistant *Pseudomonas aeruginosa*; (**B**) Wound situation after renewed hydrosurgical wound debridement using VERSAJET and application of BTM and phages for targeted therapy; (**C**) Integration of BTM after 21 days; (**D**) Definitive defect coverage with autologous split-thickness skin grafting; (**E**) Complete healing of autologous skin grafts; (**F**) 1-month follow-up.

A 69-year-old female was presented to us primarily for the care of extensive lower extremity burn injuries bilaterally. History included a domestic explosion of an ethanol stove. A total of 15% of the total body surface area was burned 2a-3°. Initial aseptic and hydrotherapeutic debridement was performed. Secondary diagnoses were arterial hypertension and diabetes mellitus. On the third day after hospitalization, the burn wounds were debrided, and the defects were covered by autologous split-thickness skin grafting. In the course of treatment, multi-resistant *Pseudomonas aeruginosa* was detected in the wound swabs without any outside signs of infection. A progressing loss of autologous skin grafts was observed due to persisting wound colonization with multi-resistant *Pseudomonas aeruginosa* (Figure 6A). All attempts at conservative wound treatment with topical applications of various aseptic solutions and water-filtered infrared light therapy (hydrosun^®^, Hydrosun Medizintechnik, Müllheim, Germany) were unsuccessful. Surgical sanitation attempts using hydrosurgical debridement with VERSAJET (Smith & Nephew, Hamburg, Germany), wound coverage with allogeneic skin grafts and renewed epifascial necrectomy and autologous split-thickness skin grafting were also unable to control the complex microbial colonization of the wound (Figure 6A). As there were no signs of sepsis or systemic infections, systemic antibiotic therapy was not indicated. Based on the imminent risk of a lower leg amputation due to exposed tendons and persisting *Pseudomonas aeruginosa* infection, all possible treatment modalities were taken into account. As the local cardiothoracic and vascular surgeons house a large bank of a variety of phages for targeted therapy, the available database was tested, and specifically matching strains were cultivated. Topical wound treatment with the cultured phages was performed to minimize the *Pseudomonas aeruginosa* colonization [38] before renewed hydrosurgical wound debridement using VERSAJET and application of BTM occurred (Figure 6B). A consistent open wound regime with daily aseptic treatment with Sulfamylon^©^ (Mafenide Acetate, Ingenus Pharmaceuticals, Orlando, FL, USA) and water-filtered infrared light therapy was used (Figure 6C). Under this treatment regime, the wound situation stabilized, so that finally, after 21 days, delamination of the BTM and autologous split-thickness skin grafting for definitive defect coverage could be performed (Figure 6D). At the time of definitive defect coverage, microbiological evidence of the *Pseudomonas aeruginosa* strain was still present. The patient did not receive antimicrobial therapy at the time of BTM transplantation. The BTM and the split-thickness skin grafts healed completely so that the patient could be discharged for follow-up burn rehabilitation treatment (Figure 6E). A 1-month follow-up showed a stable and completely healed wound situation (Figure 6F).

### 3.5. Case 5: Treatment of a Non-Healing, Chronic Lower Leg Ulcer with BTM

**Figure 7:**
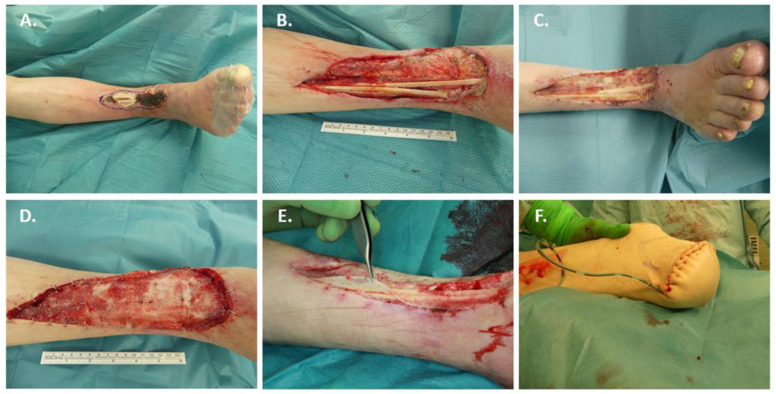
A case of a non-healing, chronic lower leg ulcer treated with BTM. A 82-year-old male was admitted with a chronic wound in the area of the right ventral lower leg: (**A**) Wound situation on admittance; (**B**) Wound situation and defect size after surgical debridement; (**C**) Wound situation after implantation of BTM; (**D**) Increasing visible vascularization of BTM after 21 days; (**E**) Failure to integrate BTM after 21 days; (**F**) Transtibial amputation according to Burghess, which healed completely.

An 82-year-old male was admitted to our emergency department. On admission, the patient reported a chronic wound in the area of the right ventral lower leg that had been present for 5 months. The wound findings were overall progressive, and a healing tendency had not been noticeable in recent months. On admission, the tendon of the tibialis anterior muscle was exposed, and the wound as a whole was fibrinous and partly necrotic (Figure 7A). Secondary diagnoses included arterial hypertension, renal anemia, and first-degree atrioventricular block. A new diagnosis on admission was chronic terminal renal failure stage V so intermittent dialysis therapy was initiated. For further diagnosis, duplex ultrasonography and computer tomography angiography of the leg vessels were performed. This revealed continued arteriosclerosis with calcified plaques within the right common iliac artery, external iliac artery and common femoral artery. Furthermore, the right superficial iliac femoral artery as well as the popliteal artery were severely stenosed or occluded. A weak contrast of the right tibiofibular trunk with exit occlusion of the right fibular artery and narrow contrast of the right posterior tibial artery including plantar branches with multiple caliber irregularities were observed. Similar high-grade vascular occlusions were also demonstrated in the left leg stromal region. Vascular surgery or radiologic intervention were deemed inappropriate because of the advanced occlusive findings. The patient had a maximum wish for preservation of the lower extremity. After radical surgical debridement and wound conditioning using vacuum dressings, the indication for defect coverage using BTM was given (Figure 7B). The vacuum dressing was changed after 14 days under general anesthesia (Figure 7C). Overall, a macroscopically visible increasing vascularization of the BTM matrix was observed (Figure 7D). On day 21, the defect was to be definitively covered by autologous split-thickness skin grafting. However, after delamination of the BTM matrix, only about 10% of the matrix was adherent and healed (Figure 7E), so that after wound refreshment with reaming of the exposed tibia, the defect was covered with MatriDerm^®^ and autologous split-thickness skin grafting. The split skin showed complete loss after removal of the overknot dressings. Due to chronic underperfusion and exhausted reconstruction and reperfusion options, the patient consented to transtibial amputation. A lower leg amputation according to Burghess was performed without complications (Figure 7F), so that the patient was admitted to the follow-up treatment with irritation-free and healed wound conditions.

## 4. Discussion

Overall, BTM application demonstrated to be a reliable and versatile reconstruction procedure for patients with multiple co-morbidities and complex wound situations. In the present study, the majority of cases were successfully treated with BTM and grafted with autologous split-thickness skin grafts. Most of the presented cases could be successfully grafted with split-thickness skin grafting on average after 3 to 4 weeks. In particular, difficult anatomical locations and infection-prone or chronically infected wounds could be healed using BTM in the present study. Furthermore, in some cases BTM showed to be robust enough to heal within the setting of partial graft loss as well. These findings go hand in hand with the published literature as BTM has been shown to be effective in the treatment of burns, necrotizing fasciitis and free flap donor wounds [34,36,39,40]. All patients in the present study underwent the described two-stage operative procedure [41]. We could not observe a complete re-epithelization after BTM treatment without the need of split-thickness skin grafting as reported elsewhere [27]. As described in other studies, the use of spray keratinocytes kit (Recell^®^) in combination with BTM could also be a promising research approach for future clinical applications [33].

One of the main advantages of BTM is the ability to convert a wound bed into a surface that is suitable for autologous split-thickness skin grafting covering of the underlying critical structures [42]. For example, the function of the underlying bone or tendon could be preserved. However, the functional orthopedic outcome, i.e., joint motion, was not further investigated in the present study and could be part of future investigations. As shown in other reports, BTM has an overall low complication profile and no donor-site morbidity. Especially in patients with multiple co-morbidities such as chronic renal failure, arterial hypertension and peripheral arterial occlusive disease, BTM showed to be a reliable and simple reconstructive option to avoid complex reconstructive operative procedures. It was described that the use of BTM in the reconstruction of lower limb complex wounds, resulted in a reduced rate of transtibial amputation [36]. Theoretically, the operative procedure could be performed under local or regional anesthesia avoiding the risks associated with general anesthesia in patients with multiple comorbidities. Thereby, intensive care treatment could be avoided, and treatment costs could be reduced. However, all of the presented cases were performed under general anesthesia. An impact on the quality of life for the patients by reducing intensive care treatment or donor-site morbidity associated for example with free flap plasties is presumed but was not further evaluated in the present study.

It was reported elsewhere that patients treated with BTM regained partial sensation over the majority of their wounds [27]. This may provide additional benefits as by regaining partial sensation, further injury of the reconstructed area could be potentially avoided as well as improve the quality of life. In the present study, the sensation of the reconstructed area was not further quantified. Further studies are needed that quantify the sensory assessment and the aesthetic outcomes, especially focusing on a long-term follow-up.

There are currently a plethora of commercial dermal skin substitutes available on the market [19]. To evaluate the value of BTM in clinical use, BTM was compared to Integra^®^ in defect coverage in a mouse model [32] and a clinical case series for defect coverage in the head and neck region [43]. Integra^®^ and BTM showed comparable results regarding infiltration with host cells, new collagen deposition and neo-vascularization, with BTM demonstrating a more extensive vascular network [32,43]. However, a greater inflammatory response was also observed in the BTM grafts at the time of integration [32].

One of the main pitfalls in the use of synthetic dermal templates is delayed vascularization and secondary infection. Li et al. observed BTM failure in infected wounds as well as radiated wound beds [27]. Especially focusing on scalp malignancies that involved burring of the outer table and radiotherapy, either partial graft failure or, in one case, complete failure of integration of BTM has been described in the literature [27]. Despite the advances in burn care and research, wound infections complicate the treatment of severely burned patients, with *Pseudomonas aeruginosa* being one of the major problematic pathogens [44]. Interestingly, the described problems of BTM loss in infected wounds could not be reproduced in the present study. Only 2 of the 20 patients had sterile wound swabs at the time of BTM application. A total of eight different pathogen strains were detected, with *Staphylococcus* ssp. and *Pseudomonas* ssp. being the most common germs. The presented findings go hand in hand with the results of a case series of defect reconstruction using BTM showing a high tolerance of the dermal scaffold against infection [45]. Furthermore, the presented case report 4 in this study also included the application of phages and BTM in the treatment of a complex wound with persisting colonization with multi-resistant *Pseudomonas aeruginosa* [38,46]. However, further clinical studies are needed to assess the long-term results of BTM and to further quantify the spectrum of application in complex wounds with a broad microbial spectrum.

Another point that should not be ignored in the clinical application of BTM is the cost factor as well as the treatment period of 21 days until definitive defect coverage by means of autologous split-thickness skin grafting. Considering the prolonged hospitalization, increased morbidity and mortality can be propagated, especially in multimorbid patients. Due to the prolonged time until vascularization and integration of the BTM matrix, an increase in treatment costs can also be expected. Therefore, it remains questionable to what extent these costs as well as the procurement costs for the BTM can be reflected in the Drug Related Disease payment system in Germany [47]. Here, further clinical experience is needed to assess the value of BTM in clinical use.

In conclusion, our findings showed that BTM application is a reliable and versatile reconstructive option, especially for patients with multiple comorbidities who are not suitable for complex surgeries or free tissue transfer for reconstruction. In the present study, infected wounds could also be healed with BTM. Although these preliminary investigations have produced promising results, significant further investigation and research are required that focus on the long-term outcomes and additional clinical applications.

## Figures and Tables

**Table 1:** Overview of an exemplary selection of dermal skin substitutes.

Name of Dermal Skin Substitute	Material	Literature
MatriDerm^®^	Bovine collagen type I, collagen type III and elastin	[21]
Dermagraft^®^	Neonatal-derived bioengineered tissue comprised of dermal fibroblasts	[22]
AlloDerm™	Donated allograft human dermis, processed to remove cells while preserving biologic components and structure of the dermal matrix	[23]
Integra^®^	Dermal component of bovine collagen type I and shark chondroitin-6-sulfate directed to the wound site and an outwardly directed silicone membrane	[24]
denovoSkin™	Hydrogel from a dermo-epidermal component after cultivation from autologous skin tissue samples	[25]
NovoSorb^®^ BTM	Biodegradable polyurethane foam with a temporary non-biodegradable polyurethane seal	[26]

**Table 2:** Defect localization of complex wounds in the study population (*n* = 20). Overall 27 complex wounds were included.

Defect Localization	Number of Wounds
Lower extremity	11
Upper extremity	6
Trunk	4
Head	3
Neck	2
Genital	1

**Table 3:** Pathogen spectrum of complex wounds with the corresponding prevalence. In the microbiological wound swabs, 9 wounds showed an isolated pathogen, whereas 9 wounds showed colonization with a mixed flora. Only 2 wound swabs were found to be sterile. (Patient population *n* = 20).

Pathogen	Prevalence
*Streotococcus agalactiae*	1
*Serratia marcescens*	1
*Corynebacterium striatum*	1
*Proteus penneri*	1
*Klebsiella pneumoniae*	1
*Enterococcus faecalis*	4
*Staphylococcus aureus* *Staphylococcus haemolyticus* *Staphylococcus epidermidis* *Staphylococcus capitis*	8 1 4 1
*Pseudomonas aeruginosa*	7

## Data Availability

The data supporting the findings of the study is available within the article.
